# Efficacy of SXN in the Treatment of Iron Deficiency Anemia: A Phase IV Clinical Trial

**DOI:** 10.1155/2019/8796234

**Published:** 2019-03-03

**Authors:** Lu Ding, Lulin Xu, Yanxia Jin, Yongchang Wei, Yunbao Pan, Safat Sattar, Yuxin Tan, Tian Yang, Fuling Zhou

**Affiliations:** ^1^Department of Hematology, Zhongnan Hospital of Wuhan University, Wuhan, China; ^2^Wuhan United Pharmaceutical Co., Ltd., Wuhan, China; ^3^Department of Clinical Oncology, Zhongnan Hospital of Wuhan University, Wuhan, China; ^4^Department of Laboratory Medicine, Zhongnan Hospital of Wuhan University, Wuhan, China

## Abstract

Shengxuening (SXN) tablet is extracted from the excrement of the silkworm and has effects on hematopoiesis. The main components of SXN are chlorophyll derivatives and sodium iron chlorophyllin (SIC). The present study aims to investigate the efficiency and safety of SXN on iron deficiency anemia. This phase IV, multicenter, open-label, randomized clinical trial was conducted in 31 hospitals in China from June 2001 to April 2002. Adults and children were randomly divided into low-dose (L-SXN), medium-dose (M-SXN), and high-dose (H-SXN) groups, respectively. The course of treatment was 1 month. Peripheral hemogram levels and iron status were examined before and after treatment. Adults in all three dose groups demonstrated a significant increase in hemoglobin (HGB) concentration. Children who received SXN treatment in medium and high doses also demonstrated increased HGB concentration. Reticulocyte counts increased at the end of treatment in the M-SXN and H-SXN adult groups and in the M-SXN child group. For both children and adults, SXN in the three dose groups was found to significantly elevate red blood cell level, mean corpuscular volume, mean corpuscular hemoglobin, and mean corpuscular hemoglobin concentration. The total effective rate in the SXN-treated group reached 84.8%. The incidence of adverse events was 4.07%. The most common side effects were nausea (2.83%), diarrhea (0.74%), and rash (0.25%). SXN was proved to be efficient and safe for adults and children with iron deficiency anemia.

## 1. Introduction

Iron deficiency anemia (IDA) affects more than 1 billion persons worldwide and is the most common cause of anemia [1]. The burden of anemia in Asia is high. The causes of IDA include decreased iron absorption, increased iron demand, low iron availability, and chronic blood loss [2]. Increasing intake of iron via methods such as food fortification and iron supplementation has been used to reduce the anemia burden. Several iron preparations, including ferrous sulphate, ferrous sulphate exsiccated, ferrous gluconate, and ferrous fumarate, are commonly used in IDA therapy, and all of these are nonheme iron formulations with adverse gastrointestinal reactions.

In traditional Chinese medicine (TCM), anemia belongs to the category of “blood deficiency.” There are two causes of blood deficiency: insufficient source and excessive blood loss. Chinese herb medicines, such as Siwu decoction [3],* Paeonia lactiflora* root [4], and Panax ginseng [5], and Chinese patent medicines such as Fufang E'jiao Jiang [6] and Sanyang Xuedai [7], which tonify both qi and blood, have effects on hematopoiesis.

**Figure 1:**
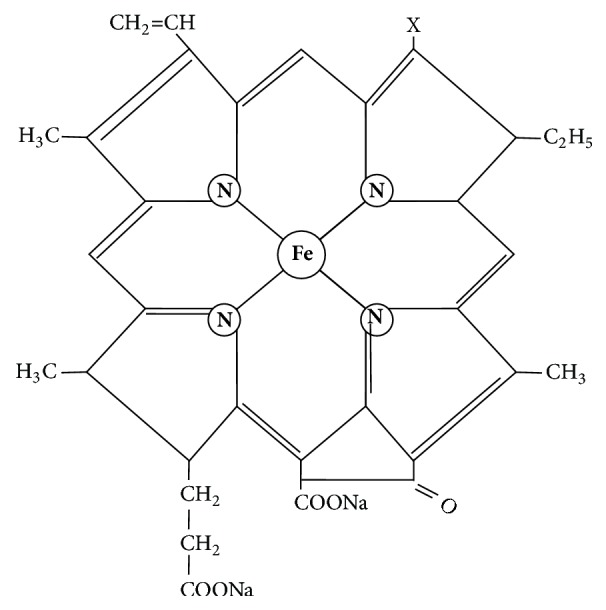
The chemical structure of SIC. SIC has a quasi-structure to heme, in which only the magnesium in the porphyrin ring has been replaced by iron.

Shengxuening (SXN) tablet, extracted from the excrement of silkworm (Bombyx mori L.) [8], has effects of replenishing qi and nourishing blood and has been used to treat anemia [9–12]. Silkworm excrement is nontoxic and has been shown to treat diabetes, fever, arthritis, and so forth [13]. In Chinese ancient medical books, including the* Compendium of Materia Medica* and* Herbal Supplements*, there are many documented uses of silkworm bombycis to treat blood deficiency and blood stasis. In addition, the methanolic extract of silkworm excrement exerts an antiproliferative effect in human colon cancer cells [14]. The main components of SXN are chlorophyll derivatives and sodium iron chlorophyllin (SIC). Chlorophyll derivatives have been shown to possess anticarcinogenic activity [15–17] and proved to be a good photosensitizer in photodynamic therapy, which is a promising modality for cancer treatment [18–20]. SIC, a water-soluble chlorophyll ferrous derivative with a heme quasi-structure (i.e., only the magnesium in the porphyrin ring is replaced by iron; Figure 1), is transported by Heme Carrier Protein 1 of the intestinal brush border. SIC can improve iron metabolism, promote the proliferation of bone marrow progenitor cells, and protect cells from oxidative stress [21–24]. The aim of the present study was to investigate the efficiency and safety of SXN in patients with IDA.

## 2. Patients and Methods

### 2.1. Participants

This phase IV, multicenter, open-label, randomized clinical trial (No. (98)ZL-040) was conducted at 31 hospitals in China from June 2001 to April 2002. Ethical approval (No. 2001ZL-005) was obtained from the Ethics Committee of national clinical drug research base of affiliated hospital of Chengdu University of Traditional Chinese Medicine. All patients recruited in the trial signed informed consent before inclusion.

A total of 2001 patients were enrolled. Inclusion criteria included (1) corresponding to the diagnosis standards of IDA; (2) compliance with deficiency of both qi and blood type (i.e., pale or sallow complexion, face or systemic edema, epigastric depression, physically and mentally fatigued, tinnitus, dizziness, pale or fat tongue, and week pulse); (3) children aged ≥6 y and <14 y and juveniles aged ≥14 y and <18 y; (4) ordinary adults aged ≥18 y and ≤65 y and elderly adults aged >65 y and ≤80 y; (5) pregnancy of 13 weeks to prenatal, 20-40 y old (late gestation group); (6) postoperative group, 18-65 y; and (7) abnormal hepatic and renal function. Patients meeting criteria (1) and (2), while meeting any one criterion from (3) to (6), and with or without criterion (7) were included. Exclusion criteria for this study included (1) anemia not caused by iron deficiency or severe IDA (hemoglobin [HGB] <30 g/L); (2) early pregnancy (before 11 weeks) and currently in the lactation period; (3) allergic constitution or allergic to SXN; (4) patients with other hematological diseases, who are psychopathic, or who have severe heart, liver, kidney, and other organ dysfunctions; and (5) primary disease that is not controlled or anemia induced by active bleeding. Approval for the study was obtained from the Ethics Committee. Three hundred sixty children (<14 y old) and 1641 adults (including juveniles, ordinary adults, elderly, pregnant, and postoperative patients) were enrolled and randomly divided into the low-dose group (L-SXN, n = 430), medium-dose group (M-SXN, n = 589), and high-dose group (H-SXN, n = 982). The list of 31 hospitals and assignment of participants are shown in Table S1.

### 2.2. Therapy

SXN tablets (Wuhan United Pharmacy) were 0.25 g/piece. The adults in the L-SXN, M-SXN, and H-SXN groups were given SXN 0.25 g twice daily (bid) orally (po), 0.5 g bid po, and 0.5 g three times daily (tid) po, respectively. The children in the L-SXN, M-SXN, and H-SXN groups were given SXN 0.25 g bid po, 0.25 g tid po, and 0.5 g tid po, respectively. The course of treatment was 1 month.

### 2.3. Observation

After 1 month of therapy, improvement in IDA of the three groups was tested, including red blood cell (RBC), HGB, reticulocyte counts, mean corpuscular volume (MCV), mean corpuscular hemoglobin (MCH), mean corpuscular hemoglobin concentration (MCHC), serum iron (SI), serum ferritin (SF), and total iron-binding capacity (TIBC) levels. Improvement in clinical symptoms (such as palpitation, pounding in the ears, headache, and light-headedness) or signs (such as pallor, glossitis, stomatitis, and angular cheilitis) was observed.

### 2.4. Efficacy

Efficacy was assessed both clinically and chemically. The criteria for total effect included (1) clinical recovery: HGB >120 g/L in men, HGB >105 g/L in women, no clinical symptoms, and normal SI, SF, and TIBC levels; (2) markedly effective: clinical symptoms improved significantly, severe anemia turned into mild anemia (at least 2 levels of improvement in the grading of anemia severity); (3) effective: clinical symptoms improved, 1 level of improvement in the grading of anemia severity; (4) ineffective: no improvement of clinical symptoms or anemia severity.

### 2.5. Statistical Analysis

Data are presented as the mean ± standard deviation in each bar graph. The *χ*2 test was used to compare enumeration data. Statistical analysis of measurement data was performed by Student's* t*-test. SPSS 19.0 statistical software (SPSS Inc., Chicago, IL, USA) was used for analysis. P < 0.05 was indicative of significant difference.

## 3. Results

### 3.1. Patients Population

A total of 2001 consecutive patients who fulfilled the inclusion and exclusion criteria were selected and enrolled in the study, including 701 men and 1300 women, with an age range of 6-80 years. Characteristics of patients in the three groups are displayed in Table 1.

### 3.2. Change in Peripheral Hemogram

**Figure 2:**
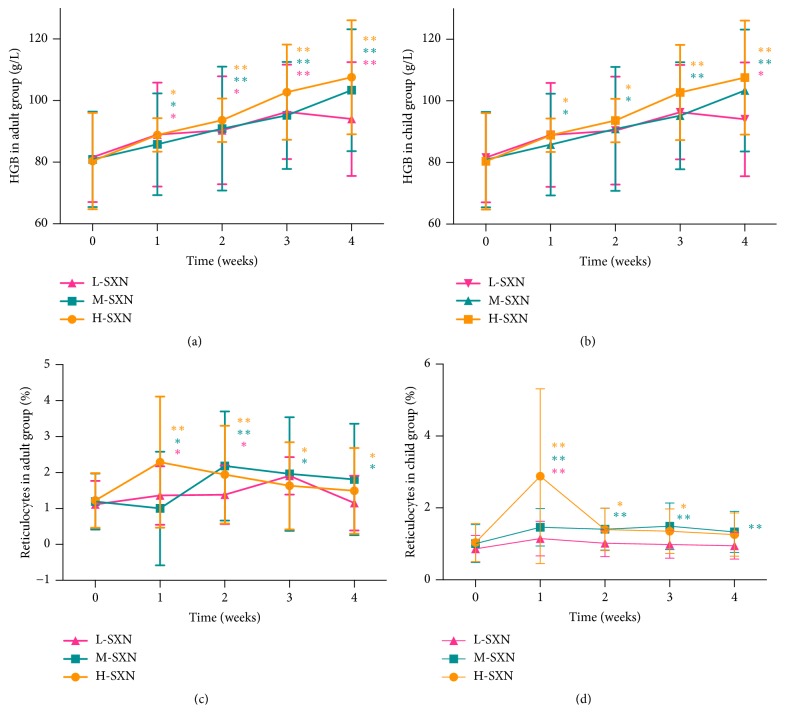
Effects of SXN on the peripheral hemogram in IDA patients. HGB increased after 1 month of SXN treatment in the adult group (a) and the child group (b). The reticulocyte counts increased at the end of treatment in the M-SXN and H-SXN adult groups (c) and in the M-SXN child group (d). *∗P* < 0.05. *∗∗P* < 0.01, compared with pretreatment.

After 1 month of SXN treatment, adults in the low-, medium-, and high-dose groups demonstrated a significant increase in mean HGB concentration (*P* < 0.01; Figure 2(a)). Children receiving medium and high doses of SXN also demonstrated increased mean HGB concentration (Figure 2(b);* P* < 0.05). The reticulocyte counts increased at the end of treatment in both the M-SXN and H-SXN groups of adults (*P* < 0.05) and in the M-SXN group of children (*P* < 0.01; Figures 2(c) and 2(d)). However, there was no significant difference in the L-SXN group of adults or in the L-SXN and H-SXN groups of children (*P* > 0.05).

As shown in Table 2, the RBC counts increased in all patients who received SXN treatment. For both children and adults, SXN in the three dose groups was found to significantly elevate the levels of MCV, MCH, and MCHC.

### 3.3. Change in Iron Metabolism

Iron metabolism was significantly improved after administering SXN (Table 3). The SI and SF levels increased, while the level of TIBC decreased obviously. The SI level increased and the TIBC declined in all groups (P < 0.01). Adults and children in the M-SXN and H-SXN groups showed evaluated SF (P < 0.01). The SF of children in the L-SXN group was not tested.

**Figure 3:**
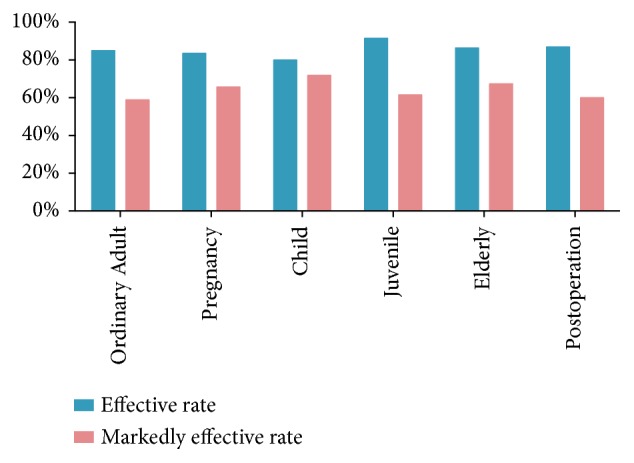
Effective rates of ordinary adults, late gestation women, children, juvenile, elderly, and postoperative patients. The effective rates in the six groups ranged from 79.9% to 91.4%.

To identify the effect of SXN treatment in patients with different conditions, we divided the 2001 patients into six groups: ordinary adults, late gestation women, children, juvenile, elderly, and postoperative. The efficacy rates in the six groups ranged from 79.9% to 91.4%, which indicates that SXN had a general effect on different kinds of patients (Figure 3).

### 3.4. Tolerability

Alanine aminotransferase (ALT) levels of 1122 cases were evaluated, both before and after treatment. None of the cases developed hepatic function abnormality. The ALT levels of 58 cases returned to normal, although they were abnormal at first. Blood urea nitrogen (BUN) levels of 1139 cases and serum creatinine (sCr) levels of 1143 cases were investigated. For patients with normal BUN and sCr levels before treatment, renal function remained in the normal range. For the 63 patients with abnormal BUN levels and 74 patients with abnormal sCr levels before treatment, their levels returned to normal after treatment (Table 4).

During the SXN treatment period, 82 patients (4.07%) experienced adverse events (Table 5). The most common side effects were nausea (2.83%), diarrhea (0.74%), and rash (0.25%). None of the patients required treatment for nausea or diarrhea, whereas one patient who experienced rash was treated by drugs. Diarrhea and rash in all cases went into remission within 3 weeks, while nausea may disappear with withdrawal. Other adverse events such as abdominal distension (0.15%) and dry mouth (0.10%) were observed. Some cases were accompanied by epigastric discomfort, stomachache, headache, angina, or acne, which were not related to SXN treatment.

## 4. Discussion

In phase IV clinical trial of adults and children aged 6 to 14 y with IDA treated in a multicenter location, HGB concentration significantly increased and iron metabolism improved with SXN treatment for 4 weeks. The efficacy rates in ordinary adults, late gestation women, children, juvenile, elderly, and postoperative patients ranged from 79.9% to 91.4%. The efficacy of SXN for improving hematological parameters, increasing iron supply, and mobilizing stored iron has been verified in an IDA rat model by regulating the iron-regulatory protein/iron-responsive element signaling pathway [25].

The structure of SIC is similar to the structure of heme, and, as a result, the effectiveness is better. The production process of SXN contains (1) extraction of chlorophyll from silkworm feces; (2) removal of the magnesium ion from the chlorophyll porphyrin ring; (3) complexing the chlorophyll derivative and ferrous iron to form SIC; (4) mixing with corn starch, dextrin, microcrystalline cellulose, low-replacing hydroxypropyl cellulose, sodium carmellose, and magnesium stearate to be made as granules; and (5) drying, compression, and film coating [25]. The fingerprint of SXN has been established to control the quality and ensure product stability (Figure S1), and X-ray diffraction has been used to verify the effective substance (Figure S2).

Previous research compared the therapeutic effect of SXN with that of ferrous gluconate in 50 IDA patients: the total effective rate of SXN reached 92%, while the total effective rate of ferrous gluconate was 32% [10]. SXN was proved to be efficient and safe in the clinical trial conducted during the period 2001-2002. In 2004, SXN was approved by the National Medical Products Administration and put into production. SXN has also been used in chronic aplastic anemia. The effective rate in 26 patients with chronic aplastic anemia patients treated with SXN and cyclosporine A reached 84.6% compared with the control group treated with stanozolol (52.6%). Except for HGB and reticulocyte levels, the levels of neutrophils and platelets also increased after treatment. The colony productivity of BFU-E, CFU-E, and CFU-GM in bone marrow significantly increased compared with the control group [12]. In a mice model of radiation-induced hematopoietic syndrome, chlorophyllin significantly enhanced the abundance of hematopoietic stem/progenitor cells and serum granulocyte-colony stimulating factor levels while increasing the survival of bone marrow cells and activating prosurvival transcription factor [26].

SXN combined with recombinant human erythropoietin significantly increased the HGB, hematocrit, SF, and transferrin saturation levels in patients with renal anemia [9]. Similar efficacy was observed in patients with anemia of maintenance hemodialysis [27]. A systematic review comprising 14 randomized controlled trials suggested that SXN was better and safer than ferrous succinate and ferrous sulphate in patients with renal anemia [28]. Although various oral and intravenous preparations of iron have been used in the correction of IDA, oral iron replacement is still the first choice in most patients because of its safety and lower cost [29]. However, the adverse reaction rate is as high as 40%, and nausea, abdominal pain, diarrhea, and constipation are common complaints. The incidence rate of adverse events observed in the present trial was 4.07%. Only 1 patient who experienced rash received medication. The other observed adverse events such as nausea and diarrhea were tolerable.

**Figure 4:**
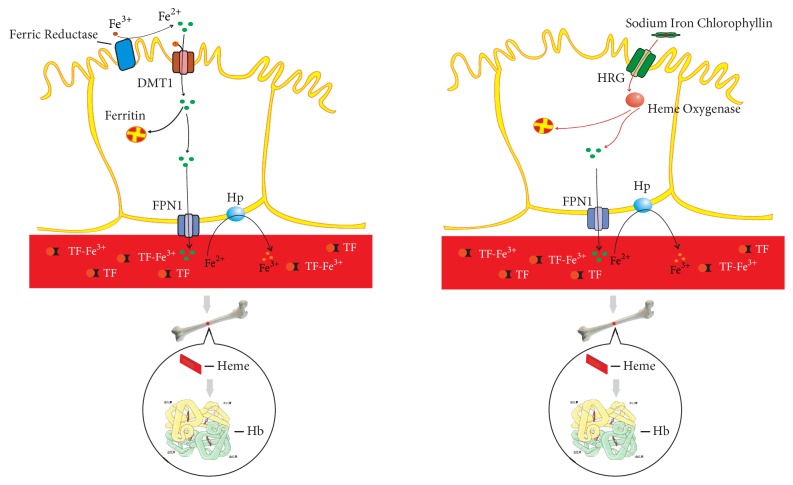
The absorption mechanism of SIC as compared with ferric iron. The mechanism of iron absorption in SIC is the same as that of heme iron, which is taken up by the heme-responsive gene (HRG).

Generally, in the lumen of the gut, dietary iron is mainly in ferric form. It must be turned into Fe^2+^ mediated by ferrireductase duodenal cytochrome b (Dcytb) before being transported across the brush border membrane. With the assistance of the membrane transporter divalent metal transporter 1, Fe^2+^ is transported into the labile iron pool in the villus enterocytes. Ferrous iron is then transferred to the circulation by FPN1, which requires hephaestin to oxidize to ferric iron in order to bind to transferrin [30]. The mechanism of iron absorption in SIC is the same as that of heme iron and is taken up by heme-responsive gene-1 or HCP1 on the apical membrane [31]. Once incorporated into the cytosol, SIC can be degraded by heme oxygenase. The released iron is excreted into circulation by FPN1 and reutilized for HGB synthesis (Figure 4). SIC remains stable under gastrointestinal conditions [32]. This can explain why SXN has high bioavailability and low gastrointestinal reactions.

**Figure 5:**
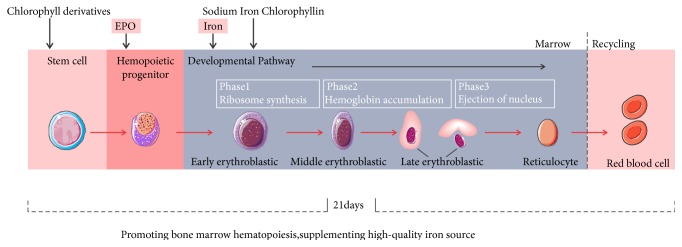
The possible mechanism of SXN in promoting hematopoiesis in IDA patients. On one hand, SIC can serve as a source of supplemental quasi-heme iron; on the other hand, chlorophyll derivatives stimulate the expression of erythropoietin.

The effects of SXN on IDA patients can be attributed to two aspects. First, chlorophyll derivatives promote the proliferation and differentiation of the precursors of the erythroid cell line by stimulating the expression of erythropoietin. Second, SIC can serve as a source of supplemental quasi-heme iron and improve iron metabolism (Figure 5). In conclusion, SXN is safe and effective for the treatment of IDA patients.

## Figures and Tables

**Table 1:** Demographic data of studied groups.

Demographic data	L-SXN	M-SXN	H-SXN	P value
Sex (n, %)				>0.05
Male	167(38.8%)	197(13.4%)	337(34.3%)	
Female	263(61.2%)	392(66.6%)	645(65.7%)	
Age (n, %)				>0.05
6~	30 (7.0%)	149 (25.3%)	181 (18.4%)	
15~	30 (7.0%)	40 (6.8%)	70 (7.1%)	
18~	250 (58.1%)	275 (46.7%)	526 (53.6%)	
65~80	205 (20.9%)	125 (21.2%)	120 (27.9%)	
Age (mean ± SD)				>0.05
Child (<14y)	9.20±1.98	9.53±2.54	9.58±2.63	
Adult (≥14y)	48.97±18.76	45.09±20.02	46.29±28.12	

**Table 2:** Effects of SXN on peripheral hemogram in IDA patients.

	Groups	n	RBC (×10^12^/L)		MCV (fL)		MCH (pg)		MCHC (g/L)	
			Pre-treatment	Post-treatment	Pre-treatment	Post-treatment	Pre-treatment	Post-treatment	Pre-treatment	Post-treatment
Adult	L-SXN	400	3.41 ± 0.67	3.72 ± 0.67^*∗∗*^	74.90 ± 8.9	79.37 ± 9.61^*∗∗*^	23.88 ± 3.66	25.43 ± 4.04^*∗∗*^	301.97 ± 43.55	321.03 ± 40.29^*∗∗*^
	M-SXN	440	3.33 ± 0.75	3.75 ± 0.67^*∗∗*^	74.76 ± 11.43	81.82 ± 11.21^*∗∗*^	24.41 ± 4.40	27.77 ± 4.27^*∗∗*^	299.29 ± 39.07	328.69 ± 43.04^*∗∗*^
	H-SXN	801	3.35 ± 0.75	3.91 ± 0.72^*∗∗*^	75.08 ± 9.99	84.41 ± 9.21^*∗∗*^	23.97 ± 4.00	27.97 ± 4.11^*∗∗*^	299.01 ± 40.83	333.76 ± 48.24^*∗∗*^
Child	L-SXN	30	3.36 ± 0.75	3.51 ± 0.45^*∗*^	74.59 ± 7.30	77.19 ± 6.22^*∗∗*^	23.87 ± 2.33	25.68 ± 1.84^*∗∗*^	287.70 ± 47.95	308.5 ± 51.06^*∗∗*^
	M-SXN	149	3.59 ± 0.74	4.18 ± 0.74^*∗∗*^	77.56 ± 7.91	86.70 ± 7.65^*∗∗*^	24.23 ± 3.46	28.97 ± 3.36^*∗∗*^	303.07 ± 35.37	334.91 ± 37.32^*∗∗*^
	H-SXN	181	3.53 ± 0.75	4.10 ± 0.67^*∗∗*^	77.86 ± 7.58	85.91 ± 9.25^*∗∗*^	24.04 ± 3.31	28.85 ± 3.11^*∗∗*^	302.95 ± 34.79	346.63 ± 42.70^*∗∗*^

*∗P*< 0.05, *∗∗P*< 0.01, compared with pretreatment.

**Table 3:** Effects of SXN on iron metabolism in IDA patients.

	Groups	n	SI (*μ*mol/L)		SF (*μ*mol/L)		TIBC (*μ*mol/L)	
			Pre-treatment	Post-treatment	Pre-treatment	Post-treatment	Pre-treatment	Post-treatment
Adult	L-SXN	400	7.37 ± 2.12	9.94 ± 4.09^*∗∗*^	11.31 ± 4.36	19.02 ± 15.46^*∗∗*^	74.58 ± 11.04	67.72 ± 2.68^*∗∗*^
	M-SXN	440	7.46 ± 2.20	12.53 ± 6.36^*∗∗*^	11.30 ± 4.79	16.09 ± 7.4^*∗∗*^	73.67 ± 6.7	63.42 ± 10.26^*∗∗*^
	H-SXN	801	7.19 ± 1.89	11.57 ± 4.62^*∗∗*^	11.48 ± 3.63	25.69 ± 23.89^*∗∗*^	74.58 ± 11.04	62.89 ± 10.74^*∗∗*^
Child	L-SXN	30	8.30 ± 0.85	8.45 ± 0.76^*∗*^	-	-	74.43 ± 8.09	71.25 ± 7.78^*∗∗*^
	M-SXN	149	7.92 ± 2.18	13.32 ± 5.16^*∗∗*^	14.33 ± 3.06	57.69 ± 47.54^*∗∗*^	78.27 ± 10.65	66.27 ± 17.44^*∗∗*^
	H-SXN	181	8.53 ± 2.84	13.24 ± 4.72^*∗∗*^	14.06 ± 2.65	56.25 ± 45.53^*∗∗*^	79.03 ± 13.08	66.85 ± 16.17^*∗∗*^

*∗P*< 0.05, *∗∗P*< 0.01, compared with pretreatment. -, untested.

**Table 4:** Safety index results before and after medication.

Blood tests	Number of tested cases	Normal pre-treatment		Abnormal pre-treatment	
		Normal post-treatment	Abnormal post-treatment	Normal post-treatment	Abnormal post-treatment
ALT	1122	990	0	58	74
BUN	1139	974	0	63	102
Cr	1143	997	0	74	72

**Table 5:** Adverse events (AEs).

Adverse event	Total number of cases	Number of cases	Correlation with drugs				Medication cases	Disappearance time	Incidence rate (%)
			Very likely	Likely	Suspicious	Impossible			
Nausea	2014	64	47	7	3	7	0	1 day~not disappear	2.83
Diarrhea	2014	15	6	5	4	0	0	1 day~2 weeks	0.74
Rash	2014	6	3	2	0	1	1	3 day~2weeks	0.25
Abdominal distension	2014	3	0	2	1	0	0	1 day~not disappear	0.15
Dry mouth	2014	2	0	0	2	0	0	1 day~not disappear	0.10
Other symptoms^*∗*^	2014	6	0	0	0	6	0	1 day~not disappear	0
Total	2014	96	56	16	10	14	1		4.07

^*∗*^ Two cases of epigastric discomfort, one case of stomachache, one case of headache, one case of angina, and one case of acne.

The cases correlated with drugs that were “very likely,” “likely,” and “suspicious” were used to calculate the incidence rate.

## Data Availability

The data was unavailable up to now, because Wuhan United Pharmaceutical Co., Ltd., was unwilling to publish primary data.

## Supplementary Materials

Supplementary Materials
*Figure S1.* The fingerprint of SXN. The fingerprint has been established to control the quality of various batches of the product. With chlorin e6 (peak S) used as a reference, a representative fingerprint of SXN showed 2 common peaks: peak 1, Fe chlorin e6; peak 2, Fe isochlorin e4.* Figure S2.* X-ray diffraction diagram of SIC. To explore the structural heterogeneity and homogeneity of the SIC, chlorophyllin, and sodium pheophorbide, three samples were ground and passed through 150 mesh to get the powder suitable for X-ray diffraction. Data were collected using Rigaku Dmax-RC X-ray diffractometer. The detection conditions were CuK*α* radiation, 50 kV pipe pressure, and 80 mA pipe flow; the scanning speed was 8°/min. Compared with the X-ray diffraction diagram of SIC, chlorophyllin, and sodium pheophorbide (data not shown), the locations of characteristic peaks are the same, but the heights of peaks are different (chlorophyllin > SIC > sodium pheophorbide (very low)), which suggests the three characteristic peaks in chlorophyllin were magnesium. The characteristic peaks of SIC (2.832 Å, 1.999 Å, 1.630 Å) indicate that magnesium ion has been replaced by ferrous iron and form the isomorphous structure.* Table S1*. Eligible subjects in 31 clinical trial centers.
