# Supplementary material for: Efficacy of SXN in the Treatment of Iron Deficiency Anemia: A Phase IV Clinical Trial
Source: Evid Based Complement Alternat Med. 2019 Mar 3;2019:8796234. doi: 10.1155/2019/8796234 (PMC6421013; doi:10.1155/2019/8796234)
Supplement: Supplementary Materials — Figure S1. The fingerprint of SXN. The fingerprint has been established to control the quality of various batches of the product. With chlorin e6 (peak S) used as a reference, a representative fingerprint of SXN showed 2 common peaks: peak 1, Fe chlorin e6; peak 2, Fe isochlorin e4. Figure S2. X-ray diffraction diagram of SIC. To explore the structural heterogeneity and homogeneity of the SIC, chlorophyllin, and sodium pheophorbide, three samples were ground and passed through 150 mesh to get the powder suitable for X-ray diffraction. Data were collected using Rigaku Dmax-RC X-ray diffractometer. The detection conditions were CuKα radiation, 50 kV pipe pressure, and 80 mA pipe flow; the scanning speed was 8°/min. Compared with the X-ray diffraction diagram of SIC, chlorophyllin, and sodium pheophorbide (data not shown), the locations of characteristic peaks are the same, but the heights of peaks are different (chlorophyllin > SIC > sodium pheophorbide (very low)), which suggests the three characteristic peaks in chlorophyllin were magnesium. The characteristic peaks of SIC (2.832 Å, 1.999 Å, 1.630 Å) indicate that magnesium ion has been replaced by ferrous iron and form the isomorphous structure. Table S1. Eligible subjects in 31 clinical trial centers. [file 8796234.f1.pdf]

Fig.S1

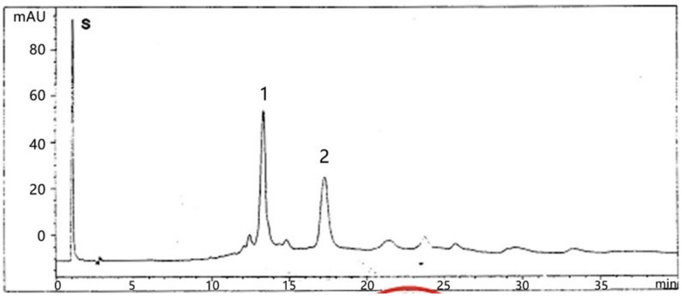

The fingerprint of SXN, with Chlorin e6 used as a reference.

Fig.S2

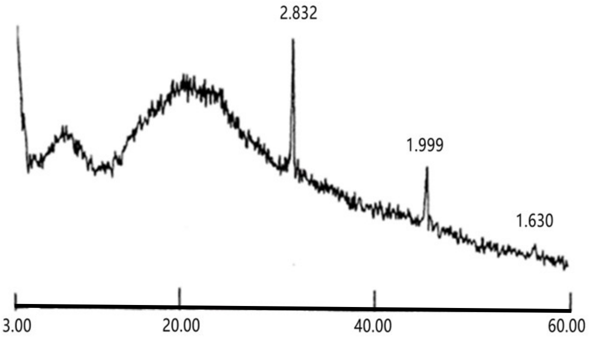

X-ray diffraction diagram of SIC.

Table S1. Eligible subjects in 31 clinical trial centers.

	Hospitals	Ordinary Adult			Pregnancy			Child			Juvenile			Elderly			Postoperation		
		L	M	H	L	M	H	L	M	H	L	M	H	L	M	H	L	M	H
1	Longhua Hospital Shanghai University of Traditional Chinese Medicine								39	41									
2	Shanghai Shuguang Hospital	20		20										20		20			
3	Yueyang Hospital of Integrated Traditional Chinese and Western Medicine	30		30															
4	Huashan Hospital of Fudan University																30		30
5	Renji Hospital Shanghai Jiaotong University School of Medicine	30		30															
6	Obstetrics and Gynecology Hospital of Fudan University					30	30												
7	Changhai Hospital of Shanghai	20		20										20		20			
8	Ruijin Hospital Shanghai Jiaotong University School of Medicine	40		40															
9	Hubei Provincial Hospital of Traditional Chinese Medicine					30	30		30	30		20	20						
10	Renmin Hospital of Wuhan University														20	20			
11	Wuhan No.1 Hospital		30	30															
12	Shashi Hospital of Traditional Chinese Medicine								30	30									
13	Yingcheng Hospital of Traditional Chinese Medicine																	20	20
14	Xiaogan Hospital of Traditional Chinese Medicine											20	20						
15	E'zhou Hospital of Traditional Chinese Medicine		30	30															

16	Xi’an Central Hospital														30	30		30	30
17	Xi’an Hospital of Traditional Chinese Medicine					30	30												
18	Shangluo Central Hospital								30	30									
19	The Second Affiliated Hospital of Chongqing Medical University														25	25			
20	Teaching Hospital of Chengdu University of Traditional Chinese Medicine	20		20															
21	The Affiliated Hospital of Southwest Medical University																30		30
22	The Affiliated Chinese Medicine Hospital of Southwest Medical University							30		30									
23	Luzhou People's Hospital					30	31												
24	Luzhou Hospital of Traditional Chinese Medicine										30		30						
25	Chengdu First People’s Hospital		25	25															
26	Zigong Hospital of Traditional Chinese Medicine								20	20								20	20
27	Zigong Third People’s Hospital													30		30			
28	Chengdu No.9 Hospital													30		30			
29	Chengdu Women’s & Children’s Central Hospital				30		30												
30	Sichuan Integrative Medicine Hospital														30	30			
31	Sichuan Province Forestry Center Hospital													20	20				
		160	85	245	30	120	151	30	149	181	30	40	70	120	125	205	60	70	130
	Total																		2001
